# Quantitative Proteomics Reveals that GmENO2 Proteins Are Involved in Response to Phosphate Starvation in the Leaves of *Glycine max* L.

**DOI:** 10.3390/ijms22020920

**Published:** 2021-01-18

**Authors:** Ling Cheng, Wanling Min, Man Li, Lili Zhou, Chuan-Chih Hsu, Xuelian Yang, Xue Jiang, Zhijie Ruan, Yongjia Zhong, Zhi-Yong Wang, Wenfei Wang

**Affiliations:** 1 College of Life Sciences, Fujian Agriculture and Forestry University, Fuzhou 350002, China; hbsdchengling@outlook.com (L.C.); Jiang_Xue1996@outlook.com (X.J.); zhijieruan@163.com (Z.R.); 2 College of Resources and Environment, Fujian Agriculture and Forestry University, Fuzhou 350002, China; wanling.m@outlook.com (W.M.); mangoliman@outlook.com (M.L.); zhoulili_1018@163.com (L.Z.); 3 Department of Plant Biology, Carnegie Institution for Science, Stanford, CA 94305, USA; cchsu@gate.sinica.edu.tw (C.-C.H.); xuelianyang@outlook.com (X.Y.); zwang@carnegiescience.edu (Z.-Y.W.); 4 Institute of Plant and Microbial Biology, Academia Sinica, Taipei 11529, Taiwan; 5 Root Biology Center, Fujian Agriculture and Forestry University, Fuzhou 350002, China; 11207024@zju.edu.cn

**Keywords:** soybean, phosphate starvation, ENO2, enolase, quantitative proteomics

## Abstract

Soybean (*Glycine max* L.) is a major crop providing important source for protein and oil for human life. Low phosphate (LP) availability is a critical limiting factor affecting soybean production. Soybean plants develop a series of strategies to adapt to phosphate (Pi) limitation condition. However, the underlying molecular mechanisms responsible for LP stress response remain largely unknown. Here, we performed a label-free quantification (LFQ) analysis of soybean leaves grown under low and high phosphate conditions. We identified 267 induced and 440 reduced differential proteins from phosphate-starved leaves. Almost a quarter of the LP decreased proteins are involved in translation processes, while the LP increased proteins are accumulated in chlorophyll biosynthetic and carbon metabolic processes. Among these induced proteins, an enolase protein, GmENO2a was found to be mostly induced protein. On the transcriptional level, *GmENO2a* and *GmENO2b,* but not *GmENO2c* or *GmENO2d,* were dramatically induced by phosphate starvation. Among 14 enolase genes, only *GmENO2a* and *GmENO2b* genes contain the P1BS motif in their promoter regions. Furthermore, *GmENO2b* was specifically induced in the *GmPHR31* overexpressing soybean plants. Our findings provide molecular insights into how soybean plants tune basic carbon metabolic pathway to adapt to Pi deprivation through the ENO2 enzymes.

## 1. Introduction

Soybean (*Glycine max* L.) is one of the most important legume corps in the world. Soybean is an excellent source of vegetable protein and oil, as well as many special nutrients, such as isoflavone, phytosterol, and saponin. Soybean is a high phosphorus (P) demand species and sensitive to low P stress [1]. Low P stress has been reported as a severe constraint affecting the final production of soybean. Soybean and other crops have developed a series of strategies to reduce the P consumption, increase the P uptake and internal P recycling, and improve phosphorus use in metabolic pathways [2]. Under phosphate-starvation environment, plants develop shallower root architecture with more and longer lateral roots and denser root hairs, as well as the exudation of organic acids and secretion of phosphatases [3]. Plants allocate more biomass to roots than shoots, resulting in a higher root/shoot ratio under low P condition. Compared with the root parts, low P triggers a set of shoot traits under both vegetative and reproductive stages, including dark-green or purple leaves due to anthocyanin accumulation, reduced leaf area and leaf numbers, and less final production. In tomato, proteomic analysis revealed that scavenging of reactive oxygen species (ROS) was induced to cope with low P content in the leaves [4]. Low phosphate reduces photosynthetic pigments and disturbs all the processes of in the photosynthetic machinery [5], as well as changes to primary carbon metabolism and other primary and secondary metabolism pathways [6,7]. 

Recent molecular and genetic analyses have revealed that a complex signaling network contributed to the relevant adaptive processes in Arabidopsis, soybean, and other crops, in which PHR1 (phosphate starvation response regulator 1) and its homologs appear to play central roles [8,9]. PHR proteins regulate multiple downstream genes by binding to the P1BS site (PHR1-binding sequence: GNATATNC) in their promoter region [8,9]. Xue et al. reported that GmPHR25 was identified as the key transcription factor in soybean, regulating downstream low-phosphate responsive genes, including *GmEXPB2* (*EXPANSIN B2*), *SPX* (*Syg1/Pho81/XPR1*), and genes encoding acid phosphatases [9]. By examination of primary and secondary metabolites, PHR1 was also identified as a key factor for metabolic reprogramming during P limitation [7]. 

However, the molecular details in low Pi stress responses remain elusive in soybean, especially in the shoot part. Recently, mass spectrometry (MS)-based proteomics have developed as one of the most powerful techniques to uncover the complex and fine-tuning signal network for plant development and response to diverse environmental factors, including nutrient-starvation conditions. To explore the phosphate-starvation tolerance mechanisms and identify the LP biomarker protein in soybean, we performed label-free quantitative mass spectrometry analysis of the soybean leaves treated with 500 μM (High phosphate, HP) and 0 μM (Low phosphate, LP) KH_2_PO_4_ for two weeks. 

## 2. Results

### 2.1. Soybean Seedlings Show Smaller Leaves and Higher Chlorophyll Concentration under LP Condition

**Figure 1:**
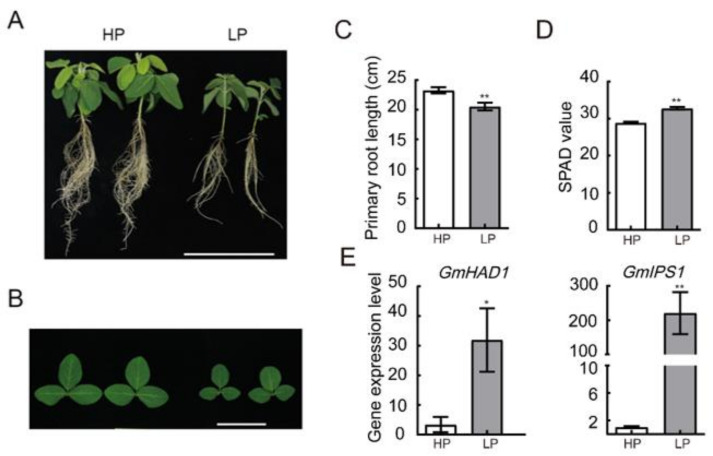
Soybean leaves under phosphate starvation. (**A**,**B**) Morphology of whole plants (**A**) and the first ternately compound leaves of 18-day-old soybean seedlings under P treatment (HP: 0.5 mM, LP: 0 mM) for two weeks in hydroponics culture. Scale bar = 20 cm in A and 10 cm in B. (**C**,**D**) Primary root length (**C**) and SPAD value (**D**) of soybean plants under HP and LP treatment, *n* = 25. (**E**) Expression level of low phosphate responsive genes *GmHAD1* and *GmlPS1*, *n* = 3. Error bars: SEM (standard error of the mean). Asterisks indicate significant difference by Student t-tests: * *p* < 0.05, ** *p* < 0.01.

After two weeks of Pi starvation (LP), the soybean seedlings’ growth was significantly repressed (Figure 1A). The shoot parts were shorter, and leaves were darker green compared with the sufficiently Pi-supplied plants (Figure 1B). The primary root length of LP-treated soybean plants (20.50 cm) was significantly shorter than the mock-treated plants (23.26 cm) (Figure 1C). Consistently, the SPAD (Soil and plant analyzer development) value of the first trifoliate leaves, which specifies the relative content of chlorophyll, was higher than that of the mock- treated plants (Figure 1D). To confirm the phosphate-starvation status, we further examined mRNA levels of two reported LP responsive genes, *GmHAD1* and *GmlPS1* [10,11]. The data clearly demonstrated the induction of these genes by this phosphate-starvation condition (Figure 1E). In agreement with these data, leaf size was smaller with an increased chlorophyll content after two weeks of phosphate starvation.

### 2.2. Homeostasis of Nutrient Elements under LP Condition

To better understand the changes of various elements in soybean plants after LP treatment, we detected element concentration by Inductively coupled plasma mass spectrometry (ICP-MS). Notably, total phosphorus (P) concentrations were significantly reduced from 7.92 mg/g to 1.26 mg/g in the leaves and from 12.48 mg/g to 9.27 mg/g in the root (Table 1). Interestingly, sodium (Na) and calcium (Ca) elements showed the same trend as P, which was reduced in both leaf and root parts (Table 1). The concentration of potassium (K) was found specifically reduced to about half the amount (from 33.22 mg/g to 17.65 mg/g) in the leaves but not in the root. In contrast, zinc (Zn) concentration was increased under LP condition in the leaves (Table 1). The concentrations of magnesium (Mg) and manganese (Mn) were decreased in the root but not shoot (Table 1). Taken together, these results suggest that phosphate starvation triggered the amount change and distribution between shoot and root of various nutrient elements.

### 2.3. LP Responsive Proteins in Soybean Leaves

**Figure 2:**
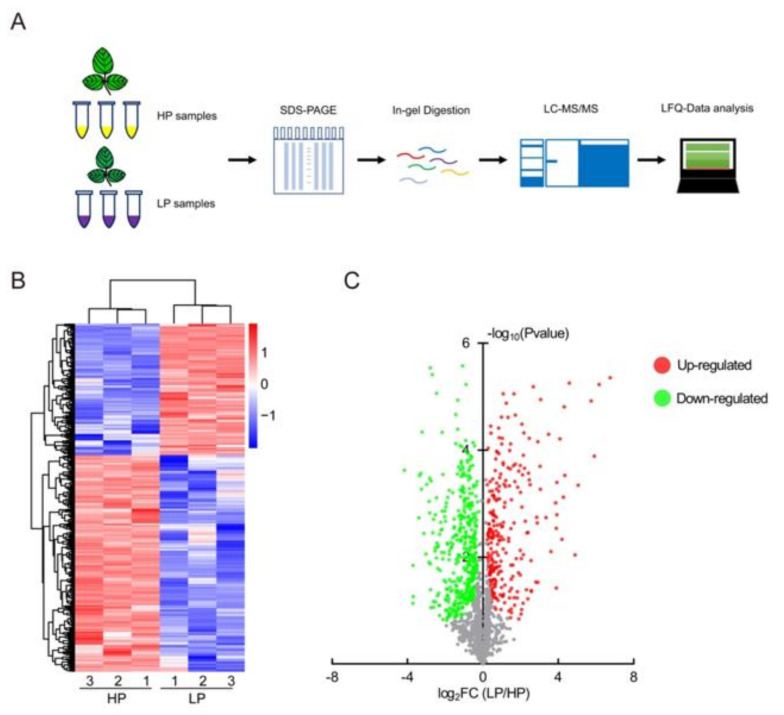
Label-free quantitative mass spectrometry analysis of soybean leaves under phosphate starvation. (**A**) Flowchart for the identification of LP regulated proteins by LC-MS/MS. (**B**) Heatmap of expression profiles for differential proteins. The color scale represents average log signal intensity values. LFQ intensity that significantly decreased is displayed in blue, while increased is in red. (**C**) Volcano plot of differentially expressed proteins between LP and HP leaves. Red dot indicates LP increased proteins, green dot indicates LP decreased proteins and grey dot represent proteins with no significant difference between LP and HP; FDR < 0.05; S0 value = 0.1.

In this study, we sought to identify soluble protein signatures in soybean leaves that may be used as biomarkers for low phosphate response and monitoring. To profile the leaf proteome triggered by low phosphate, we isolated total soluble protein from three samples of the first trifoliate leaves after HP and LP treatment. The protein isolation workflow including the following mass spectrometry analysis steps is illustrated in Figure 2A. To identify the differentially expressed proteins, extracted soluble proteins were digested in gel and analyzed by liquid chromatography-tandem mass spectrometry (LC-MS/MS), using higher-energy collisional dissociation (HCD). Using MaxQuant and Perseus software, label-free quantification (LFQ) was performed to identify differentially expressed proteins (Figure 2B). From a total of 4192 identified proteins, 267 increased proteins and 440 decreased proteins with false discovery rate (FDR) < 0.05 were sorted out and displayed by the volcano plot (Figure 2C). 

### 2.4. Phosphate Starvation Induced Changes of Glycolysis Processes in Soybean Leaves

**Figure 3:**
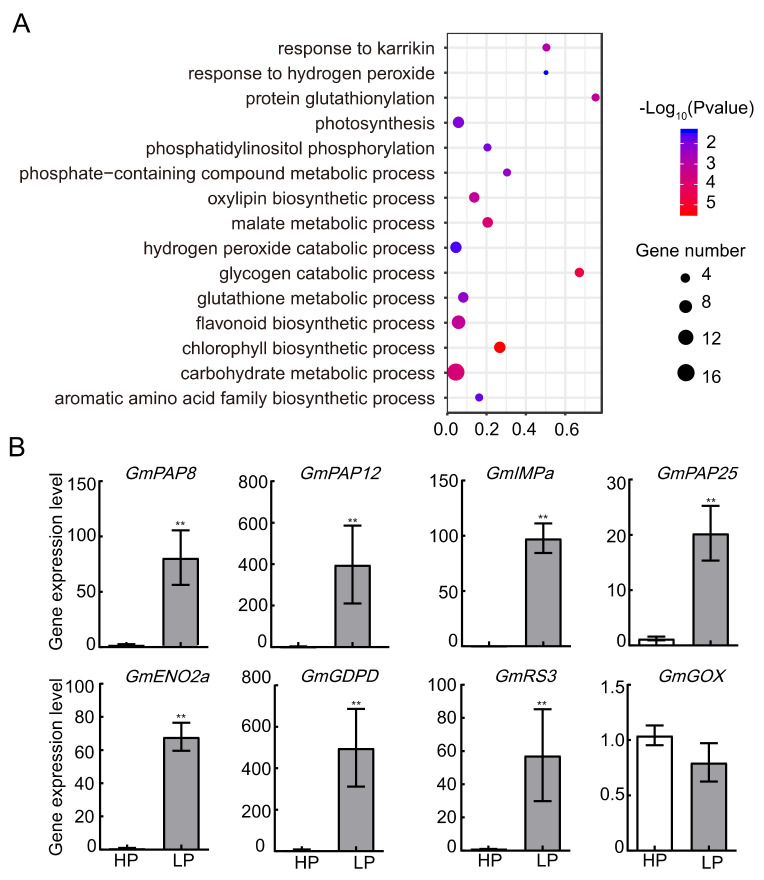
GO and qPCR analysis of LP-induced proteins. (**A**) Functional categorization of LP-induced proteins based on biological processes. Bubble colors represent the corrected P-value, and the bubble sizes indicate the number of genes. (**B**) The qPCR analysis of LP-increased genes identified in LFQ analysis. Error bars: SEM. Asterisks indicate significant difference by Student t-tests:, ** *p* < 0.01.

To better understand how LP regulates biological processes in soybean leaves, Gene Ontology (GO) analysis was performed. The most enriched GO categories of the LP-induced proteins were chlorophyll biosynthetic process, glycogen catabolic process, malate metabolic process, and carbohydrate metabolic process. Consistent with our SPAD value measurement (Figure 1D), chlorophyll biosynthetic process-related genes were highly enriched from the LP-induced genes (Figure 3A). To investigate the transcriptional patterns, eight upregulated proteins were chosen for qRT-PCR analyses on mRNA level (Figure 3B). The mRNA levels of most selected proteins (7/8) were found to be significantly increased by LP, including one enolase (GmENO2a, Glyma.09G153900), three soybean purple acid phosphatases (GmPAP8, 12, and 25), one glycolate oxidase (GOX), a Myo-inositol monophosphatase (IMPa), and a raffinose synthase (RS3). Enolases are involved the glycolytic pathway, catalyzing the reversible dehydration of 2-phospho-glycerate (2PG) to phosphoenolpyruvate (PEP) [12]. Previous studies revealed that a large number of GmPAP (Purple acid phosphatase) genes were induced or enhanced by Pi starvation in soybean [13], which were believed to play important roles in P acquisition and recycling in plant. Seven GmPAP proteins were identified from our samples, among which three PAP genes, GmPAP8, GmPAP12 and GmPAP25, were found to be dramatically induced (Table S1, Figure 3B). RS3 protein was found to be increased by LP (Figure 3B), which was reported to catalyze the process that converts sucrose and galactinol to raffinose and myo-inositol. Three myo-inositol monophosphatase (IMP) were identified as increased proteins (Table S1, Figure 3B), which are required for the synthesis of myo-inositol from inositol (1,4,5)-trisphosphate [14]. Plastid localized GDPD (Glycerophosphodiester phosphodiesterase) proteins were reported to regulate cellular phosphate homeostasis under phosphate starvation in Arabidopsis and rice [15,16]. We found a homolog of GDPD protein in soybean was increased by LP (Table S1). The overexpressing *glycolate oxidase* (*GOX*) in chloroplasts accumulates both hydrogen peroxide (H_2_O_2_) and glyoxylate [17]. *GmGOX1′*s mRNA level was not changed (Figure 3B). However, its protein amount was greatly increased in LP condition (Table S1). That is, we detected the transcription levels of LP-upregulated genes were mostly consistent with their change on protein level.

### 2.5. Phosphate Starvation Reduced Changes of Translational Machinery in Soybean Leaves

**Figure 4:**
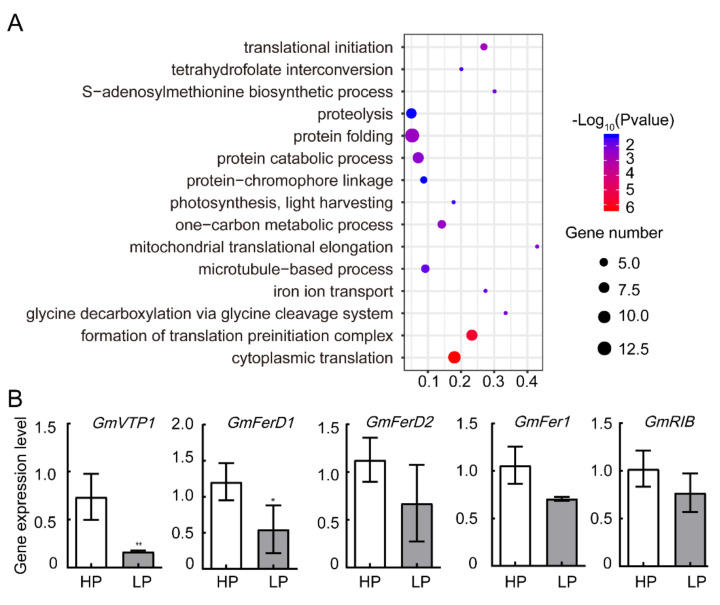
GO and qPCR analysis of LP-reduced proteins. (**A**) Functional categorization of LP-reduced proteins based on biological processes. Bubble colors represent the corrected *p*-value, and the bubble sizes indicate the number of genes. (**B**) The qPCR analysis of LP-decreased gene identified in LFQ analysis. Error bars: SEM. Asterisks indicate significant differences by Student t-test: * *p* < 0.05, ** *p* <0.01.

For downregulated proteins, four processes-related GO terms were found to be enriched, including cytoplasmic translation, formation of translation preinitiation complex, protein catabolic process, and protein folding (Figure 4A). A V-type proton ATPase protein (GmVTP1, GLYMA_14G151400) was found as a downregulated protein (Table S1) and its mRNA level was correspondingly decreased by LP stress (Figure 4B). Ferredoxins transfer electrons from the photosystem I to multiple redox-driven enzymes involved in the assimilation of carbon and nitrogen [18]. Ferredoxin protein levels were decreased under LP treatment while their coding genes *GmFerD1* decreased about half and *GmFerD2* had no significant change (Figure 4B). Ferritin proteins play key roles in iron homeostasis and protection against iron-mediated oxidative stress. *Arabidopsis Ferritin 1* (*AtFer1*) gene was reported to be directly upregulated by the Phosphate Starvation Response 1 (AtPHR1) transcription factor [19]. However, our result showed that GmFER1(GLYMA_03G050100) protein was decreased by LP (log_2_FC = −2.79) (Table S1), while its mRNA level was not significantly changed (Figure 4B). Interestingly, about a quarter of downregulated proteins (109/440) are translation processes-related proteins, including ribosomal proteins, translation initiation factors, and elongation factors (Table S1). The 40S ribosomal protein GmRIB (Glyma.03G086400) was decreased by LP (log_2_FC = −1.44), while the mRNA level was not changed significantly (Figure 4B). In agreement with these data, LP reduced a large amount of protein levels in protein translation machinery.

### 2.6. GmENO2a and GmENO2b Genes Were Regulated by LP Treatment

**Figure 5:**
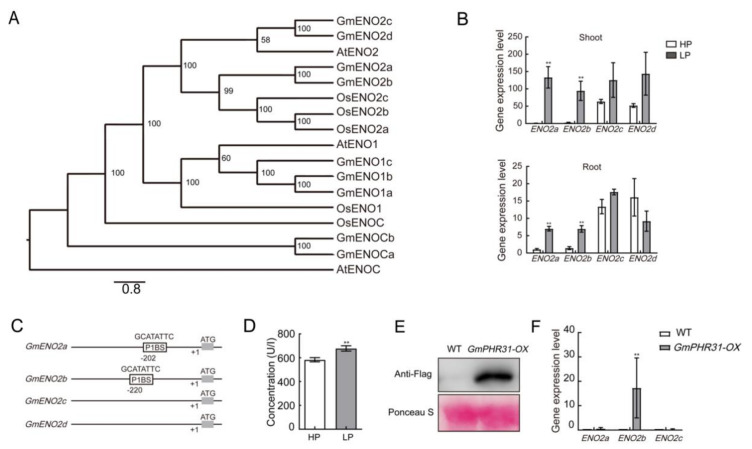
Enolase was induced by phosphate starvation. (**A**) Phylogenetic analysis of the ENO proteins in Arabidopsis, rice, and soybean. At, *Arabidopsis thaliana*; Os, *Oryza sativa*; Gm, *Glycine max*. (**B**) The mRNA levels of soybean *ENO2* genes in response to phosphate starvation. (**C**) Diagram showing the structure of the *GmENO*2 promoters. P1BS motifs were boxed. (**D**) Enolase concentration was increased by LP treatment in soybean leaves. (**E**) Immunoblot of GmPHR31 protein level. Ponceau stain was used as loading control. (**F**) Expression level of *GmENO2b* increased in *GmPHR31* overexpression line. Error bars: SEM. Asterisks indicate significant differences by Student t-tests: ** *p* <0.01.

Enolase, also known as phosphopyruvate hydratase, is a metalloenzyme responsible for the catalysis of the conversion of 2-phosphoglycerate (2-PG) to phosphoenolpyruvate (PEP) [20]. The 14 enolase encoding genes were identified from soybean genome (Table S2). To evaluate the evolutionary relationship of ENO proteins in soybean, multiple protein sequences of ENO family proteins from rice, Arabidopsis, and soybean were aligned and analyzed (Table S2). The results showed a high degree of similarity among ENO proteins from these plants. Soybean ENO proteins were clearly divided into three subfamilies, ENO1, ENO2, and ENOc subfamilies (Figure 5A). By comparing with the amino acid sequence of AtENO2 family protein, four homologs of AtENO2 were named as GmENO2a-d according to the evolutionary homology (Figure 5A) [12]. Three GmENO1 proteins were found and named as GmENO1a, GmENO1b, and GmENO1c. According to our MaxLFQ results, GmENO2a protein level was dramatically upregulated in LP (Table S1). 

We detected the transcription level of *GmENO2* genes in the shoot and root of soybean with Pi treatment. *ENO2a* and *ENO2b* were significantly increased by LP treatment in both shoot and root (Figure 5B). Interestingly, the transcription level of *GmENO2c* and *GmENO2d* was significantly higher than that of the other two genes under HP condition, but did not respond to the LP treatment (Figure 5B). Comparing the structure of soybean enolase gene, only *GmENO2a/b* but not *GmENO2c/d* genes have P1BS motif (Figure 5C), not the other enolase genes. In addition, the concentration of enolase was determined by ELISA analysis and was found to be significantly upregulated by LP (Figure 5D). To test the hypotehsis that the expression of *ENO2* genes are regulated by the PHR transcription factor, we constructed overexpressing, stable transgenic lines of GmPHR31, and the GmPHR31 protein level was detected by immunoblot (Figure 5E). By qPCR anaylsis, the expression level of *GmENO2b* was significantly increased in the *GmPHR-OX* plants (Figure 5F). These data suggested that *GmENO2a* and *GmENO2b* are LP responsive genes and *GmENO2b* is promoted by overexpression of *GmPHR31* transcription factor in soybean.

## 3. Discussion

### 3.1. Phosphate Starvation Disturbs the Homeostasis of a Series of Nutrient Elements

In response to the phosphate deficiency, concentrations of Ca and Na declined in both leaves and root, concentrations of Zn and potassium were disturbed in the leaves only, while Mg and Mn were decreased in the root, specifically (Table 1). The interaction between Pi and Zn homeostasis was recognized and studied at the physiological and molecular levels. Negative relationships between Zn and P concentration have been reported in many crops, including wheat, maize, and lettuce [21,22,23]. A recent report by Jain et al. (2013) showed that Zn starvation causes a repression and induction of the expression of *PHT1;1* in roots and shoots, respectively[24]. However, in the soybean, the molecular detail of how phosphate starvation induces the Zn accumulation in the leaves but not in the root remains unclear. Similar to our results, potassium is found to be specifically reduced in the leaves in phosphate-starved Arabidopsis and poplars [25,26]. In Arabidopsis, phosphate starvation has been correlated to a modification of iron distribution and to an increase of iron content in tissues [27]. However, under our condition, the total iron concentrations were not affected in either shoot or root. *AtFER1* gene was reported to play an important role in the homeostasis between iron and phosphate, which is directly promoted by PHR and PHL transcription factors [19]. However, soybean FER1 protein level was decreased in leaves while its transcriptional level was not changed under our condition (Figure 4B). One possible explanation is that the soluble proteins we extracted may not have presented the total FER1 protein or Gm*FER1* may not be regulated the same way as that in Arabidopsis, which need further experimental data.

### 3.2. Phosphate Starvation Tuned down the Translation Machinery in Soybean Leaves

Similar to the previous report [28], under phosphate-deficient condition, soybean reduced the P concentration in the shoot and limited shoot development (Figure 1, Table 1). Higher root-to-shoot ratios are commonly observed in the LP-stressed plants. Changes in gene expression levels were identified from shoot parts of Arabidopsis and soybeans [28,29]. Phosphate starvation promoted a large amount of LP responsive genes mainly by PHR family transcription factors, through P1BS binding motif in their promoter region [9]. In Arabidopsis, 42 ribosomal protein genes were found to be downregulated on the transcriptional level after 72 h of low phosphate treatment [30]. Our results provided a direct evidence as to how LP regulated shoot growth on the protein level. Large numbers of translationally related proteins were decreased in the soybean leaves. There were no significant changes on the mRNA level of GmRIB protein (Figure 4B), which probably explained the previous transcriptome analysis that did not find this downregulation on translation processes in the early stage of phosphate starvation [29]. 

### 3.3. Phosphate Starvation Regulates GmENO2 to Alter the Balance of Carbon Metabolisms

Previously, Wu et al. reported that two genes for PEP carboxylase and malate dehydrogenase were repressed during Pi starvation and *PK* (*Pyruvate Kinase*) gene, which converted PEP to pyruvate, was upregulated [30]. As a key enzyme in the glycolysis for PEP generation, *AtENO2* plays an important role in a series of growth and developmental processes, including seed development, pollen germination, and floral organogenesis [12]. In addition, *AtENO2* was reported to be involved in the response to freezing stress [31] and salt stress [32]. Interestingly, *AtENO2* works as a multifunctional gene encoding two proteins, the full-length AtENO2 and truncated version AtMBP-1 (c-Myc binding protein 1-like) [33,34]. The *eno2* mutant showed impaired function in glycolysis and constitutive developmental defects, which are correlated with a strong reduction in enolase activity but not AtMBP-1 transcript abundance [12]. Unlike the ENO1 and ENO3 proteins located in chloroplast and cytoplasm, ENO2 proteins were found to locate in cytosol and nuclei [35]. ENO2 interacts with bZIP57 to control the cytokinin content to regulate seed development [31]. Unlike Arabidopsis, there are four members of the soybean ENO2 subfamily and their functions are still unclear. Our work showed that phosphate starvation triggered higher activity in soybean leaves (Figure 5C). *ENO2c* and *ENO2d* are highly expressed in phosphate-sufficient condition and did not respond to phosphate starvation. Meanwhile, *ENO2a* and *ENO2b* were induced by the LP in the leaves of soybean, probably through the P1BS binding motifs. 

## 4. Conclusions

Our LFQ analysis revealed a proteomic response of soybean leaves to Pi deprivation. LP limited shoot growth by tuning down the number of proteins involved in translation processes and mobilized a series of fundamental metabolic and biological processes by raising proteins related to chlorophyll biosynthetic and carbon metabolic pathway. Our data showed LP induces *GmENO2a* and *GmENO2b* levels and overexpressing *GmPHR31* induces *GmENO2b’s* expression in soybean. The current results indicate a key role of ENO enzyme in integrating a basic carbon metabolic pathway into LP response in soybean leaves. Our findings will provide the basis for further functional characterizations of these responsive proteins and the molecular mechanism of LP adaption and will benefit the biomarker development for early detection of LP stress in soybean.

## 5. Materials and Methods 

### 5.1. Soybean Growth and Phenotype Analysis

Soybean seeds (*Glycine max* var. *Williams 82*) were germinated in the wet vermiculite for three days and then transferred into modified half-strength Hoagland’s nutrient solution (HP, 500 μM KH_2_PO_4_; LP, 0 μM, pH = 5.8) for another two weeks. The Hoagland’s solution was renewed every three days. Seedings were harvested for proteomic profiling, gene expression analysis, and nutrients content analysis, respectively. The 18-day-old soybean seedlings were photographed, and the primary root lengths were measured by ImageJ. The SPAD value for chlorophyll concentration of each leaf was measured using a chlorophyll meter (SPAD-502 Plus; Konica Minolta, Chiyoda City, Japan). Then, 25 plants were measured in each treatment.

### 5.2. Nutrients’ Element Detection

Leaf and root tissue of soybean seedlings after HP and LP treatment were used for the total element quantitative measurements. After drying at 65 °C for 3 days, samples were weighed and digested in concentrated H_2_SO_4_. The concentrations of nutrient elements were determined by ICP-MS (7900 Mass Spectrometer; Agilent, Santa Clara, CA, USA).

### 5.3. Quantitative Real-Time PCR Analysis

The total RNA was isolated by the RNA Simple Total RNA Kit (TIANGEN, Beijing, China) and synthesized to cDNA by the prime Script RT-PCR kit (Takara, Japan). The quantitative RT-PCR was performed with TB GreenPremix Ex TaqII (Takara, Japan) by QuantStudio 6 Flex Real-Time PCR System (Life Technologies, Carlsbad, CA, USA). Soybean *UBQ13* gene was used as an internal control, and the relative expression levels were calculated by 2^−ΔΔCT^ method. All primer sequences are listed in Table S3.

### 5.4. Protein Extraction, in Gel Digestion, and LC-MS/MS Analysis

Three biological replicates from HP- and LP-treated leaves were used for soluble protein extraction. Total soluble protein was isolated from the first trifoliate leaves of soybean leaves. Fresh leaves were homogenized to slurry by a cold pestle, then filtered by a two-layer Miracloth (Millipore, Burlington, MA, USA). Supernatant was mixed with Extraction Buffer (0.5 M Tris-HCl, 10% SDS, 20% (*w*/*v*) Glycerin, 2% (*w*/*v*) β-mercaptoethanol) with a ratio of 1:1 and heated at 95 °C for 10 min. The debris was removed by centrifugation at 12,000 g for 15 min. Protein concentration was determined with bovine serum albumin (BSA) as a standard. Protein samples were loaded into a precast gradient SDS PAGE gel (NuPAGE Novex 4–12% ((wt/vol) acylamide in buffer) Bis-Tris Protein Gels; Invitrogen, Carlsbad, CA, USA) and digested with trypsin. The resulting peptides were analyzed by HCD LC-MS/MS using an Orbitrap Fusion mass spectrometer (Thermo Fisher, Waltham, MA, USA). Survey scans were acquired in the Orbitrap MS using a mass resolution of 140,000. Six MS/MS scans were acquired in the Orbitrap for each survey scan.

### 5.5. Label-Free Quantitative Analysis

Raw intensities were extracted and normalized using standard settings with the LFQ selected by MaxQuant [36], followed by statistical analysis using Perseus software [37]. Missing values were replaced by imputation according to normal distribution to simulate low abundance values in a typical MS experiment. Differentially expressed protein (FDR <0.05, S0 value set as 0.1) between LP and HP samples were selected as the LP responsive proteins for the following analysis. We used the Novogene tool (https://magic.novogene.com/customer/main#/tool-ngs/) to perform bi-directional clustering analysis of all different genes in the samples. According to the expression levels of the same gene in different samples and the expression patterns of different genes in the same sample, the heatmap was obtained through correlation distance and average linkage.

### 5.6. GO Term Enrichment and Statistical Analysis

Functional analysis was performed using the DAVID (version 6.8) online service (https://david.ncifcrf.gov/) to evaluate the function of the protein identification [38]. All retrieves’ terms were selected by REVIGO (http://revigo.irb.hr/) [39] and visualized by the R Programming Language [40].

### 5.7. Enolase Enzyme-Linked Immunosorbent Assay

The first trifoliate leaves were homogenized in ice-cold phosphate buffer saline (PBS) solution (v:v = 1:10) with a pestle. Supernatant was filtered by Miracloth (Millipore, USA) and measured with Enolase Enzyme-linked immunosorbent kit (Quanzhou Ruixin, China). The supernatant was loaded to the sample plate and mixed with horseradish peroxidase (HRP)-labeled antibody and incubated at 37 °C for 60 min. After rinsing with washing solution for 1 min, substrate was added and incubated in the dark at 37 °C for 15 min. It was quenched with the stop solution and then the optical density (OD) value of each sample was measured at a wavelength of 450 nm. A linear regression equation was obtained based on the OD values of the measured standards (200, 100, 50, 25, 12.5, 6.25 U/I), and the OD values of the samples were substituted into the equations to calculate the sample concentration. 

### 5.8. Protein Alignment and Phylogenetic Tree Construction

Sequences of ENO2 family proteins from *Arabidopsis thaliana* (At), *Oryza sativa* (Os), and *Glycine max* (Gm) were blasted from Phytozome (https://phytozome.jgi.doe.gov/pz/portal.html#) (Figure 5A). All sequences were aligned and truncated by Geneious [41]. Clustalx1.83 was used to save the matched sequence in NXS format. Matrix parameters were modified according to the Bayes model, a reconstructed Bayesian inference (BI) tree on MrBayes, and run for 1,000,000 generations in total [42]. The phylogenetic relationships of the Enolase family of them were reconstructed by using Bayes method. AtENOC was used as an outgroup.

### 5.9. Plasmids’ Construction and Plant Transformation

Full sequence of *GmPHR31 (Glyma.19G146600)* was cloned from cDNA of soybean, and then overexpression vector *pFGC5941-35S::GmPHR31-3×Flag* was constructed by subcloning GmPHR31 to the *pFGC5941:35S::3×Flag* using *Asc I* restriction site. The overexpressing transgenic lines were generated through transforming into *A. tumefaciens strain* EHA105 [43]. The soybean genotype *Glycine max* var. *Williams 82* was used as the explant for stable transformation, as described previously [44].

### 5.10. Immunoblotting

Cotyledons of 5-day-old seedlings were harvested and crushed in liquid nitrogen. Powder tissues were dissolved in 2×SDS loading buffer (0.5M Tris-HCl, 10% SDS, 20% glycerol, 2% β-mercaptoethanol, 1% bromophenol blue), incubated at 95 °C for 10 min, and then centrifuged (5 min, 13000 rpm). We loaded 20 μL of supernatant, separated by 8% SDS-PAGE, and transferred it to a polyvinylidene fluoride (PVDF) membrane (Bio-Rad, USA). After transfer, the PVDF membrane was blocked overnight by 5% skimmed milk. The membrane was then incubated with primary anti-Flag antibodies for 3 h at room temperature (Diluted 1:3000; TransGen Biotech, China), washed three times in 1×PBST (1 L, 8 g NaCl, 0.2 g KCl, 3.58 g Na_2_HPO_4_•12H_2_O, 0.24 g KH_2_PO_4_, 0.1% Tween-20), incubated for 1 h with anti-mouse-HRP secondary antibodies, and washed five times in 1×PBST. The blot was developed by Chemiluminescence SuperSignal West Dura (Thermo Fisher, USA).

## Figures and Tables

**Table 1:** Concentration of nutrient elements measured by ICP-MS.

Element	Shoot				Difference	Root				Difference
	HP		LP			HP		LP		
	Average (μg/g)	STDEV	Average (μg/g)	STDEV		Average (μg/g)	STDEV	Average (μg/g)	STDEV	
P	7923.521	918.434	1257.768	141.069	**	12,481.891	2378.997	927.414	101.639	**
Mn	76.327	12.644	58.764	5.906		60.531	11.647	19.167	0.665	**
Fe	242.162	66.500	200.234	36.175		454.848	179.639	372.961	58.063	
Al	28.941	8.830	24.709	7.169		76.987	11.649	132.375	47.597	
Mg	4362.344	882.239	3938.867	219.738		10,985.741	1456.902	2002.847	235.717	**
B	35.347	9.818	40.156	9.748		20.797	6.392	19.227	10.973	
Na	128.089	13.426	75.672	5.761	**	469.947	50.065	223.533	12.833	**
K	33,216.546	7712.960	17,645.706	3582.965	*	56,132.335	2661.463	55,887.859	1526.117	
Ca	3425.835	558.822	2025.138	88.246	*	600.464	12.329	501.423	6.413	**
Zn	65.079	6.197	89.514	1.508	**	91.868	20.228	79.114	12.090	
Co	0.299	0.063	0.355	0.007	*	4.503	1.281	2.588	0.458	
Ni	10.580	0.405	7.871	0.665		12.686	3.689	9.897	1.430	
Cu	7.818	0.919	9.491	0.486		18.194	1.318	20.503	1.239	*
Rb	3.707	0.719	2.415	1.051		6.205	0.645	5.185	1.281	
Sr	7.142	1.575	4.279	0.690		4.549	0.505	4.096	0.585	
Mo	30.259	13.767	25.377	1.535		182.092	24.899	160.434	30.439	

Seedlings were treated with HP and LP, and the shoot and root parts were harvested for ICP-MS. Three biological replicates were used for each sample. Asterisks indicate significant differences between LP and HP samples by Student t-test: * *p* < 0.05, ** *p* < 0.01.

## Data Availability

Publicly available data sets were analyzed in this study. These data are available via ProteomeXchange with identifier PXD023343 (Username: reviewer_pxd023343@ebi.ac.uk, Password: DEu0ui30).

## Supplementary Materials

The following are available online at https://www.mdpi.com/1422-0067/22/2/920/s1. Table S1: Differentially expressed proteins in soybean leaves under LP condition. Table S2: Alignment of the amino acid sequences of ENO proteins in Arabidopsis, rice, and soybean. Table S3: List of primers used in this study.

## Abbreviations

ELISA	Enzyme-linked immunosorbent assay
ENO	Enolase
GDPD	Glycerophosphodiester phosphodiesterase
GO	Gene ontology
ICP-MS	Inductively coupled plasma mass spectrometry
LC-MS/MS	Liquid chromatography followed by tandem mass spectrometry
LFQ	Label-free quantification
P1BS	PHR1-binding sequence
SPAD	Soil Plant Analysis Development
