# Supplementary material for: Quantitative Proteomics Reveals that GmENO2 Proteins Are Involved in Response to Phosphate Starvation in the Leaves of Glycine max L
Source: Int J Mol Sci. 2021 Jan 18;22(2):920. doi: 10.3390/ijms22020920 (PMC7831476; doi:10.3390/ijms22020920)
Supplement: Supplementary file 1 [file ijms-22-00920-s001.zip › supplementary files/Table S3 List of primers used in this study.docx]

Table S3: List of primers used in this study.

| **primer name** | **Forward Primer** | **Reverse primer** | **Process** |
| --- | --- | --- | --- |
| *qGmHAD1* | GTGACAACTGGGTCGTCGATGAT | CAGGTATAACTCTGGGGTGCAAG | Real-time qPCR |
| *qGmIPS1* | TGATAGTACCGGATTATCAAGG | CTGATAGGCATAGTTTGCAG | Real-time qPCR |
| *qGmPAP8* | GCATCATGGTAACACCGAAGA | TCCACCTCCACTTGTTATAAAG | Real-time qPCR |
| *qGmPAP12* | TCCATTATAGCAGTCCCTTTTGTG | TGGTTCCTCTACCCAGCCAAT | Real-time qPCR |
| *qGmIMPa* | TGTGTCTCCATCGGTCTTACA | CCTTTCCCACGAATTCCAGT | Real-time qPCR |
| *qGmPAP25* | AGCCATGGATGTGGAATGTGG | TGGAAGCCGAATAGGGAGTAGT | Real-time qPCR |
| *qGmENO2a* | TCGGGAAAGGTGTTCTCAAG | CCCCATTCATTAACAGTTCCA | Real-time qPCR |
| *qGmGDPD* | TCGGAGTGACTTCAAGATGC | TTGATGGCTCTCATTCTTCG | Real-time qPCR |
| *qGmRS3* | AACTGAAGCAATATCCCTTG | GACACCCTTGTAGCCAAAAT | Real-time qPCR |
| *qGmGOX* | CAGCAGCTGGCACAATCATG | TCTCACAAGCTGAGCAACCAC | Real-time qPCR |
| *qGmVTP* | GGCCCTCGTCTTCTCCTGTA | CCGCAATAATCAAACCGTAAA | Real-time qPCR |
| *qGmFerD1* | CTACTCATGCAGGGCTGGTTC | TTGTGGGTTTCAATGACAACG | Real-time qPCR |
| *qGmFerD2* | GAACTTCCCTACTCGTGCAGG | AGGTTGAGCAACACAGGTGAG | Real-time qPCR |
| *qGmFer1* | GCTGATGAGTCCGAATCTGC | TTGGCAAGTCCCTTGAGAGC | Real-time qPCR |
| *qGmRIB* | GGCGAGGAAGCCACATAGAG | CTGGCGACCAAGCTCATACT | Real-time qPCR |
| *qGmEno2b* | AGCCACAGGAGTGGTGAAAC | TGGTTGTACTTGGCAAGCCT | Real-time qPCR |
| *qGmEno2c* | ATGGCGAGTCACAGAAGTGG | GCACCAAGCTCCTCCTCAAT | Real-time qPCR |
| *qGmEno2d* | GAGCTCCATGCAGGTCTGAG | TAGTAGGGTTCAACGGGGGT | Real-time qPCR |
| *qGmUBQ13* | GTGTAATGTTGGATGTGTTCCC | ACACAATTGAGTTCAACACAAACCG | Real-time qPCR |
